# Understanding Smallholder Pigkeepers’ Awareness and Perceptions of African Swine Fever and Its Control Measures in Ukraine

**DOI:** 10.3390/pathogens13020139

**Published:** 2024-02-03

**Authors:** Lidiia Moskalenko, Katja Schulz, Vitalii Nedosekov, Kerli Mõtus, Arvo Viltrop

**Affiliations:** 1 Institute of Veterinary Medicine and Animal Sciences, Estonian University of Life Science, 51014 Tartu, Estonia; kerli.motus@emu.ee (K.M.); arvo.viltrop@emu.ee (A.V.); 2 Institute of Epidemiology, Friedrich-Loeffler-Institut, Federal Research Institute for Animal Health, 17493 Greifswald-Insel Riems, Germany; katja.schulz@fli.de; 3 Department of Epizootology, National University of Life and Environmental Science of Ukraine, 03041 Kyiv, Ukraine; nedosekov06@gmail.com

**Keywords:** African swine fever, smallholder pig farmers, participatory epidemiology, control and preventive measures, biosecurity, acceptability

## Abstract

African swine fever (ASF) has posed a significant threat to Ukrainian pig farming since its identification in 2012. In this study, recognising the pivotal role of pigkeepers in disease control, we conducted ten focus groups involving 52 smallholders across eight regions in Ukraine. Using participatory methods, we revealed their awareness of ASF signs, transmission routes, preventive measures, and the perceptions of stakeholders involved in ASF control. Furthermore, we identified the smallholders’ acceptance of eradication and restriction measures, the perceived impact of zoning consequences, and their main sources of ASF information. Smallholders identified fever and skin haemorrhage as the most indicative signs of ASF and highlighted rodents as a primary transmission concern. Disinfection was seen as the most effective measure for preventing the introduction of ASF. Pigkeepers who perceived their stakeholder role in ASF control showed more trust in themselves and veterinarians than in central veterinary authorities. Farm-level ASF eradication measures were generally accepted; however, culling within the protection zone was least accepted, with economic losses listed as the most impactful consequence for pigkeepers. For ASF information, pigkeepers favour web searches and veterinarians, as well as traditional media and word-of-mouth news. This study provides valuable insights into refining the ASF communication strategies in Ukraine.

## 1. Introduction

African swine fever (ASF) is a viral haemorrhagic disease that affects domestic pigs and wild boars and has serious socioeconomic consequences in affected countries. When ASF outbreaks occur, the impact can be significant, affecting not only the pig industry, but also other sectors of the economy [1]. The first incursion of the African swine fever virus (ASFV) into Ukraine dates back to 1977, when it was introduced to the southwestern part of the country, presumably through food waste from ships arriving at the Odesa city seaport. The outbreak was successfully contained within six months by implementing strict control measures and eradicating the entire pig population in the affected region [2]. In July 2012, an ASF outbreak was reported in a small holding in the Zaporizhzhia region, in the southeast of Ukraine. The most likely route of virus introduction was swill feeding [3]. No further outbreaks were reported until January 2014, when the virus was first identified in wild boars in the Luhansk region. This region borders the Russian Federation, where the last cases of ASF in wild boars were reported in December 2013. After the first detection of ASF in 2014, the disease spread throughout the domestic pig and wild boar populations, eventually affecting all administrative regions of Ukraine by the end of 2017. Ukrainian ASF surveillance identified an ongoing circulation of ASFV in both the wild boar and domestic pig populations [4].

In less than a decade, the pig sector of Ukraine has undergone significant transformations, characterised by a decrease in the number of domestic pigs within smallholdings. As of 2020, the pig population is estimated to be 3.3 million in the commercial sector and 2.43 million in smallholdings. While domestic pig herds in the smallholder sector are distributed throughout the entire country, their number is greatest in the western regions of Ukraine [5]. Ukrainian law defines smallholders as individuals within families whose products are used for subsistence within the household and sold at marketplaces or directly to other persons if the production exceeds family consumption needs [6]. Therefore, these pigs also provide an additional source of income for households. Pigkeepers must identify and register their households and pigs in the Unified State Register of Animals in Ukraine no later than 60 days after the animal is born [7]. The Ukrainian government implemented an order stipulating measures for the prevention and eradication of ASF. In the case of a suspicion of ASF in pigs, the pigkeeper must inform the veterinary office of their respective district or city. The chief veterinary officer is then responsible for initiating disease eradication measures within the affected farm and the surrounding region [8]. In response to the quarantine measures enforced to control and prevent ASF, the state provides compensation to smallholder pigkeepers [9], with payments applicable for pigs registered in the state animal register [10].

Throughout the ASFV epidemic during the last century in Ukraine, tackling the challenge of ASF control was particularly complex for smallholdings. This complexity stems from the frequent outbreaks that occur predominantly within these farms [4]. When addressing such epidemiological scenarios, the primary emphasis should be placed on control measures targeting the factors that contribute to the spread of ASFV. In this context, the virus seems to have been transmitted to new areas primarily because of the uncontrolled movement of live pigs and pig meat between villages [11,12]. Factors associated with human behaviour have certainly played an important role in many countries during ASF outbreaks, particularly in regions where smallholder farming is prevalent [13,14,15,16]. Pigkeepers play an important role in controlling and preventing ASF in domestic pigs through the implementation of biosecurity measures. Consequently, farmers’ willingness to accept ASF control and eradication measures holds significant importance in ensuring the effectiveness of the control system. A fundamental aspect of this is that farmers have a strong understanding of the basic characteristics of the disease. Following the introduction of ASF into Ukraine in 2012, international campaigns have been implemented in various regions to promote the awareness of ASF, its transmission pathways, and adequate biosecurity measures [17,18,19].

This study targeted smallholder pigkeepers who primarily keep pigs for their own consumption. In recent ASF epidemics in Europe, controlling the disease in the domestic pig population has been particularly challenging in countries with substantial smallholder pig sectors [20,21]. This may be due to a lack of awareness of ASF among smallholders. Another potential factor contributing to this situation might be inadequate communication between the authorities responsible for disease control and the pigkeepers. This communication gap may lead to misunderstandings between authorities and pigkeepers, ultimately reducing the acceptance of control measures and compliance with them.

In this study, we aimed to investigate the awareness, perceptions, and attitudes of smallholder pigkeepers towards ASF and its control measures in Ukraine. More specifically, we focused on the awareness of ASF’s clinical signs, transmission routes, and preventive measures. We aimed to reveal the pigkeepers’ awareness of the stakeholders involved in ASF control and their level of trust in these stakeholders. We also sought to explore how smallholder pigkeepers accept eradication measures and restrictions during ASF outbreaks. In addition, our objective was to determine which sources of information on ASF were used and deemed important by pigkeepers.

## 2. Materials and Methods

### 2.1. Organization of Focus Groups (FGs)

The target group of this study was smallholder farmers rearing up to ten pigs on their farms in Ukraine. The study participants were recruited using convenience sampling. Participants were contacted via phone calls, personal contacts, or publicly available contacts from Facebook groups dedicated to swine welfare. During the invitation process to join the focus group (FG), all participants were informed about the study’s organisation, study aims, and voluntary participation, including their ability to drop out without any reason at any time. When organising the focus group discussions (FGDs), the goal was to include smallholder pigkeepers from different parts of Ukraine, with three to seven participants per group. Prior to their first implementation in the field, FGDs were conducted and study questions administered at the Estonian University of Life Sciences by a volunteer FG consisting of students with a veterinary background. The participants’ feedback was incorporated into the FGs’ further implementation.

Meetings were conducted from May to October 2021 on the property of one participant. The FGs were moderated in Ukrainian and Russian by the same female facilitator. Meetings were conducted by the facilitator, who was trained in the methodology applied. Every participant provided positive oral consent to audio recording and anonymous use of the gathered data for scientific publications. To keep the FGs anonymous, participants’ names and demographic information, such as age and sex, were not recorded. The facilitator then transcribed the meeting recordings.

### 2.2. Tasks of the FGD and Employed Participatory Tools

The meetings were organised into eight tasks. Each aimed at investigating various aspects related to ASF control in smallholder farms where domestic pigs are kept. To quantify the awareness, acceptability, and affinity of pigkeepers, we employed proportional piling and visualisation tools, drawing inspiration from previous studies [22,23,24,25]. Furthermore, we introduced a 10-bead scoring tool during Tasks 4 and 6, allowing the group to collectively score each listed item based on the questions presented. For this, 10 glass beads per listed item were provided to the participants, which they then allocated based on their evaluations. Qualitative findings from the recorded FGDs were descriptively incorporated into the analysis.

### 2.3. Task 1: Awareness of ASF Signs in Domestic Pigs

In this task, participants were asked to list signs that might lead them to suspect ASF within the herd. Using proportional piling, they were asked to express their opinions on which of these signs would lead them to suspect ASF in the herd. The group was instructed to reach a consensus on the allocation of 100 glass beads, with the distribution reflecting the perceived indicative value assigned to each sign.

Through the use of participatory tools and discussions, the facilitator regularly enquired into participants’ statements and decisions to confirm their accurate understanding of the task and foster further discussions.

### 2.4. Task 2: Awareness of ASFV Transmission Routes

The group was tasked with listing all possible transmission routes for the introduction of ASFV into smallholdings. Then, they were asked to express their opinion on which of these transmission routes posed the highest risk of ASF introduction into smallholdings (proportional piling).

### 2.5. Task 3: Awareness and Attitude towards ASF Preventive Measures

The group was instructed to list preventive practices that could be applied to avoid the introduction of ASFV into a smallholding. Then, participants were asked to express their opinions on the effectiveness of the listed measures at preventing the introduction of ASF (proportional piling).

**Figure 1:**
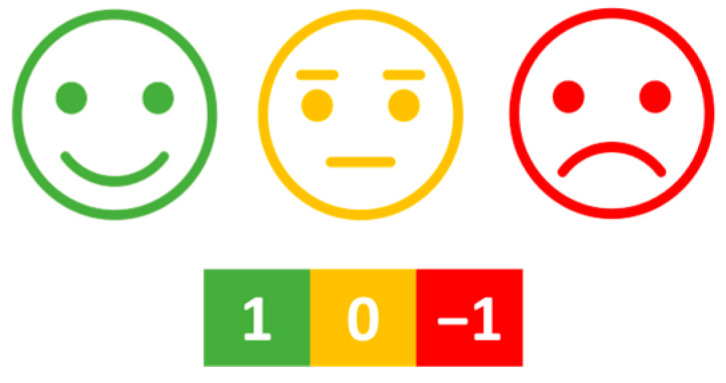
Visualization tool that was used to illustrate pigkeepers’ personal opinions.

Subsequently, the participants were asked to express their personal opinions on how much they liked implementing these measures, setting aside their effectiveness. Each individual’s opinion was expressed using face emojis (Figure 1), accompanied by verbal explanations for the given evaluation. The emojis were colour-coded, green signifying a positive response, yellow for neutral, and red for a negative response, to visually represent each participant’s stance. For semi-quantitative analysis, each emoji was assigned a rank.

### 2.6. Task 4: Perception of and Trust towards Stakeholders Involved in ASF Control

For this task, participants were asked to name all the institutions and stakeholders contributing to the compliance, execution, and surveillance of control and preventive measures for ASF. Participants explained how they perceived the role of each listed stakeholder in ASF control. If ‘pigkeepers’ were not initially listed as stakeholders, the facilitator suggested their inclusion, recognising that participants might not have self-identified in relation to the question. This prompted further discussion in which participants could decide whether to include themselves. Then, they were asked to express their opinion on which of these stakeholders played a larger role in preventing and fighting ASF in the country (proportional piling).

Subsequently, participants were asked to express their trust in the capabilities of the listed stakeholders to fulfil their respective roles in ASF control. Using a 10-bead scoring tool, ten glass beads were designated to each stakeholder on the list. The group assigned scores to reflect their collective opinion on a scale ranging from zero (indicating complete mistrust) to 10 (complete trust).

### 2.7. Task 5: Acceptability of Farm-Level ASF Eradication Measures

The facilitator presented a list of four ASF eradication measures applied to outbreak herds and ensured uniform understanding among participants.

- Farm quarantine;
- Culling of all pigs on the farm;
- Destroying the feed and bedding materials on the farm;
- Cleaning and disinfection of the farm.

The participants were then invited to express their personal acceptance of each measure (face emojis) and to verbally articulate the rationale behind their assessments.

### 2.8. Task 6: Acceptability of Measures Applied in ASF Restricted Zones

The facilitator presented a list of three restriction measures applied during zoning in the case of an ASF outbreak and ensured uniform understanding among participants.

- Culling of all pigs in the protection zone;
- Restrictions on moving pigs in the protection zone;
- Restrictions on trading live pigs and pork products in the protection and surveillance zones.

The participants were asked to express their collective acceptance of each listed restriction measure (10-bead scoring tool).

### 2.9. Task 7: Consequences of ASF Zoning

The group was provided with information indicating that restricted zones were established around ASF-affected farms, which led to restrictions on the movement of pigs and trade of pigs and pork products. Participants were then asked to identify the consequences for pigkeepers when their farms fell within these restricted zones. Then, they were asked to allocate glass beads to express their opinion regarding which of these consequences had the greatest impact on pigkeepers (proportional piling).

### 2.10. Task 8: Sources of Information about ASF

In the last task, participants were asked to name all the sources from which they had received or expected to receive information about ASF. Then, were instructed to indicate the relative importance of the information sources (proportional piling).

### 2.11. Data Management and Analysis

After each FG meeting, the collected data were entered into Microsoft Excel (2019) spreadsheets and translated into English. After analysing the audio recordings, the entire dataset in the Excel file was rechecked to avoid possible insertion mistakes. The listed items with similar contents were then merged into a single entity by creating a common notation (see the example in Table 1).

Numerical data from the division of 100 glass beads were used to analyse the proportional piling tool. The sum of the weighted proportional piling score $S_{i}$ for each listed item i was computed as follows:

(1) $$S_{i}=\sum_{j=1}^{10}{GB}_{ij}\cdot \frac{N_{j}}{N_{total}},$$

where ${GB}_{ij}$ indicates the number of glass beads assigned to the listed item i by one group j; $N_{j}$ is the number of items listed by one individual group *j* during the task; and $N_{total}$ is the sum of all listed items in one task by all 10 groups.

For the analysis of face emojis, a numerical value was allocated to each response: +1 for positive, 0 for neutral, and −1 for negative (see Figure 1). The numerical rank values assigned by all voting participants to each listed item across the ten meetings were then summarised. Finally, the sum was divided by the total number of voting participants to obtain the average emoji score.

For the analysis of the 10-bead scoring tool, the mean score value for each listed item was calculated by summing the scores given by participants from all focus groups in which it was listed.

## 3. Results

### 3.1. Recruitment of Participants

**Figure 2:**
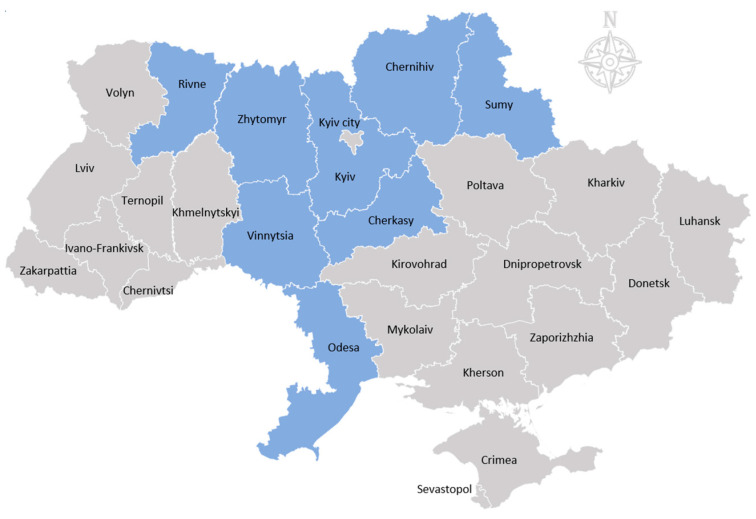
Map of Ukraine with regions in blue depicting where FGs were conducted.

In total, ten meetings were conducted with three to seven participants per FG. The pigkeepers (*n* = 52), 13 male and 39 female, were recruited from eight administrative regions (Table 2, Figure 2). The estimated age of the participants ranged from 20 to 80 years, with approximately 6 aged under 40 years old, 7 between 40 and 49 years old, 19 between 50 and 59 years, and 20 over 60 years. None of the participating pigkeepers had directly experienced the confirmed outbreak, although one participant was affected by the restrictions imposed in an ASF surveillance zone.

### 3.2. Awareness of ASF Signs in Domestic Pigs

Among the 16 listed signs, fever was considered the most indicative sign that would incline pigkeepers to suspect ASF in the herd, (*n* = 10). Skin haemorrhage was ranked as the second most indicative sign (*n* = 8), with three groups mentioning that this could also be a characteristic sign of other diseases, such as erysipelas. Additionally, two groups that did not mention skin haemorrhages revealed that they lacked knowledge of the characteristic signs of ASF, yet would assume a viral infection in the case of high fever, and accordingly consult a local veterinarian. The other parameters are summarised in Table 3.

### 3.3. Awareness of ASFV Transmission Routes

Among the 18 listed transmission routes, rodents were identified as posing the highest risk for ASFV introduction to the pig herd (*n* = 5). Pigkeepers cited the easy access of rodents to pig holdings, transport vehicles, fields, forests, and barns where feed was stored. Shoes were ranked as the second riskiest route (*n* = 8). Pigkeepers explained that the virus could be transmitted by the dirty footwear of visitors, such as veterinarians, animal dealers, and pig owners. Transport vehicles were designated the third riskiest route (*n* = 7). During the discussions, all vehicles entering the village or territory of the pigkeepers’ holdings were considered potential risk factors. The fourth riskiest route was human-related transmission and activity (*n* = 6). For both transport vehicles and humans, participants mentioned dealers who buy pigs and travel long distances as well as veterinarians who use the same pair of shoes and clothes for all their client visits throughout the day. Feed was identified as the fifth riskiest route of ASFV introduction (n = *6*). Half of the groups cited using commercially sourced grain, which could potentially be exposed to multiple contacts, while the other half mentioned grains grown in their own fields; both could be at risk of rodent encounters during storage, as noted by pigkeepers. Beyond grains, the participants discussed other supplements to the pig diet, such as commercial feed, boiled vegetables, and herbs. One group mentioned providing kitchen leftovers. Wild animals, including wild boars, ranked eighth in terms of risk factors (n = *5*). It was mentioned that wild animals may still find ways to enter villages or access the territories of pigkeepers, including their fields. Table 4 summarises the other transmission routes.

### 3.4. Awareness and Attitude towards ASF Preventive Measures

Among the 18 listed preventive measures (Table 5), disinfection of pig premises ranked first in terms of effectiveness in preventing the introduction of ASF into herds (*n* = 10). This was moderately favoured due to the chemical nature of the disinfectants, which could have an unpleasant smell and adverse effects on the lungs and eyes (e.g., chlorine). Some participants disclosed a preference for lime treatment on their walls because it helps maintain a clean environment for animals. Access bans for people were ranked as the second most effective measure (*n* = 6). This was highly favoured for its ability to isolate animals from potential sources of contamination and its ease of implementation. Changing clothes and shoes was ranked as the third most effective preventive measure (*n* = 6). Pigkeepers indicated that they typically have separate shoes for working with animals and in the field, but that having separate clothes is less common. The use of a ‘vaccine’ as a preventive measure was mentioned once by a group that claimed to have documents from a veterinarian that their pigs were vaccinated against ASF and classical swine fever (CSF) viruses.

### 3.5. Perception of and Trust towards Stakeholders Involved in ASF Control

The participants listed 14 stakeholders who contributed to the development, enforcement, and implementation of ASF control measures. Pigkeepers were assigned the highest rank based on their perceived role in controlling ASF (*n* = 10). From the discussions, it appeared that pigkeepers were recognised as responsible for implementing measures to ensure pig welfare and adhering to instructions provided by veterinarians and authorities.

Pigkeepers expressed a high level of trust in their fellow pigkeepers, considering themselves reliable in carrying out their roles in ASF control. They also displayed considerable trust in the district veterinary hospital and local veterinarians (both private and official), recognising their competence and expertise in the field. At the same time, the level of trust in the central veterinary authority or village administration was relatively lower. During the group discussions, the pigkeepers’ level of trust appeared to be linked to the perception that the highest level of veterinary authority and governmental bodies merely issued directives without sufficient engagement in or consideration of farmers’ concerns. Furthermore, many participants expressed concerns that authorities at various levels might make efforts to avoid providing compensation for depopulated pigs. A summary of the other stakeholders is provided in Table 6.

### 3.6. Acceptability of Farm-Level ASF Eradication Measures

The most widely accepted eradication measures were the cleaning and disinfection of the farm, followed by farm quarantine (Table 7). Both were seen as justified measures with the highest impact on virus eradication in the infected area, without requiring additional effort or investments. Culling all animals ranked third in terms of acceptance by the participants. While it was regarded as a justified measure, it was less favoured because of the established emotional bond with the pigs. Destroying feed and bedding materials on farms was the least accepted measure. Most groups claimed that the destruction of commercial feed and additives for pigs leads to economic losses. Expressed arguments highlighted the fact that commercial feed is typically stored in its original bags in separate barns, which reduces the probability of contamination. Some participants expressed a preference for having a document confirming feed contamination to justify its destruction. In addition, one group suggested that uncontaminated grains could be used for human consumption. The destruction of bedding materials was considered a justified measure for eradicating the disease.

### 3.7. Acceptability of Measures Applied in ASF Restricted Zones

Culling all the pigs in the protection zone was the least accepted control measure. Six groups evaluated this measure positively, mentioning its importance for preventing further spread of the virus. Others questioned the necessity of culling if pigs were healthy and showed no clinical signs of infection. The explanations for the lowest scores were linked to financial losses, potential stress, feelings of useless effort, and wasted time. Restrictions on moving animals and trading live pigs and pork products were broadly accepted by pigkeepers (Table 8).

### 3.8. Consequences of ASF Zoning

During this task, 12 consequences arose from being in a zone with ASF restrictive measures applied in the event of an outbreak. Concerning the perceived impact of these consequences on pigkeepers, economic loss ranked first (*n* = 7), with participants discussing its importance in impacting the economy of households. The precautionary slaughter of pigs for consumption ranked third (*n* = 7). During the discussions, the participants disclosed that they would most likely process slaughtered pigs into heat-treated canned meat as soon as information on the establishment of restriction zones reached them (word-of-mouth news) to precede the culling of animals by veterinary authorities. The participants explained these actions as measures to prevent economic loss, as they did not believe that they could receive fair compensation for the culled pigs, emphasising that they would never slaughter a sick animal. At the same time, participants from one group argued that their neighbours could slaughter sick animals for heat-treated canned meat because the disease was not zoonotic. The other results are presented in Table 9.

### 3.9. Sources of Information about ASF

Of the 11 listed sources providing information about ASF, sources accessed via Google web search were attributed the highest importance (*n* = 8), with participants discussing their quick and easy access to relevant information about ASF. Traditional media, including television news and radio, ranked third (*n* = 9) for staying informed of the broader picture of ASF at the national level. Spoken communication (word-of-mouth news) was the fourth most important source (*n* = 8) for staying updated on local news, as discussed. The accuracy of this information was questioned unless it was related to nearby outbreaks or restrictions imposed. Then, veterinarians ranked fifth (*n* = 3), with participants highly valuing the qualifications of local veterinarians. Furthermore, pigkeepers indicated that more sources in Ukraine with detailed explanations of ASF’s signs, transmission routes, preventive measures, biosecurity procedures, surveillance, and legal frameworks are needed. A summary of the other sources of information about ASF that were discussed is presented in Table 10.

## 4. Discussion

It has been shown that a participatory approach can improve communication among different disciplines, foster mutual understanding, and consequently facilitate effective disease control [26,27,28]. Notably, participatory methods have been successfully employed in the evaluation of ASF surveillance [22,29] and to appraise the attitudes of farmers towards ASF control in Northern Uganda [30] and Tanzania [31], as well as in Lao PDR [32] and the Philippines [33]. Several participatory studies addressing ASF control have recently been conducted in Europe. The perceptions of farm managers regarding ASF and its control have been studied in Estonia [34]. Further insights into ASF control and surveillance in wild boar have been gained by studying the perceptions of hunters in Estonia, Latvia, and Lithuania [24,25,35].

Given the often limited resources and access to veterinary services, active involvement in implementing control measures and adherence to biosecurity practices by pigkeepers can greatly reduce the chances of ASF transmission. By doing so, pigkeepers not only protect their own livestock, but also contribute significantly to the broader containment of the disease. In this study, we aimed to gain insights into the awareness, perceptions, and attitudes of smallholder pigkeepers related to ASF and its control measures in Ukraine.

Regarding disease recognition in pigs, pigkeepers demonstrated adequate awareness of the early signs of ASF, which is expected to empower them to promptly report their suspicions to their veterinarians. Non-specific ASF signs such as fever, loss of appetite, and lethargy were mentioned as important indicators of ASF in pigs and were given a high ranking in the assessment. Similar results were obtained in a recent questionnaire study conducted across five regions of Ukraine, in which most respondents selected fever and lethargy as clinical signs of ASF in pigs [36]. Nevertheless, there is room for improvement, as several participants highlighted a gap in their understanding of the characteristic signs of ASF such as skin haemorrhage. This underscores the importance of veterinary authorities continuing their efforts to reach every pigkeeper to ensure that essential knowledge regarding ASF is effectively delivered and comprehended. In our study, participants did not name ASF postmortem findings. We assumed that this could be explained by how the question was presented to the participants. A broad term ‘sign’ was used without specifying ante- or postmortem signs. It may be that the pigkeepers might not connect the term ‘sign’ with lesions in internal organs or that they do not know this sign, or they understood the question to be related only to living animals. Several relevant ASF transmission routes that present a risk of virus introduction to domestic pigs were listed by the participants. Nevertheless, the relatively less important routes (mechanical vectors, e.g., rodents, airborne transmission) were given a higher ranking than the routes of higher epidemiological relevance (direct contact with a wild boar, indirect modes of transmission, e.g., swill feeding). It can be assumed that pigkeepers’ opinions concerning the high risk of ASF introduction by rodents were influenced by national legislation regulating ASF control, which highlights deratisation as a crucial measure required in outbreak farms and within the protection zone [8]. There is no evidence that ASFV is spread over long distances by droplets or air [37]. Simultaneously, within a short range (such as within a pigsty), the transmission of ASFV through the air has been demonstrated [38]. The participants frequently cited air as a possible transmission route for ASFV. During discussions, pigkeepers often drew parallels between ASF and COVID-19, leading to the assumption that ASFV may also be capable of airborne transmission. This suggests that they might have been confused about COVID-19, thinking that ASFV, as a virus, might have the same routes of transmission. In turn, swill feeding, which is considered one of the main possible routes for ASFV introduction into pig herds, particularly in smallholdings [11,39,40,41], was rarely brought up by the participants during discussions. In a study by Muñoz-Gómez et al. [33], swill feeding was found to be a common practice on smallholder pig farms in Ukraine. The lack of awareness of the risks related to swill feeding among the study participants is highly worrying and emphasises the need to distribute relevant information to smallholders and ensure that the information truly reaches the target groups. None of the FGs named a theoretically important route of ASFV transmission: ticks. This may be due to the fact that the soft tick Ornithodoros verrucosus currently inhabiting the southern regions of Ukraine [42] is unlikely to be capable of transmitting ASFV [43]. Therefore, measures against ticks are not included in the national legislation regulating ASF control in Ukraine. Consequently, pigkeepers are not provided with information on this aspect by authorities, and in turn, they cannot name it as a transmission route. In general, the results demonstrate an important knowledge gap among smallholder pigkeepers concerning the routes of ASFV transmission, which could potentially affect the implementation of preventive measures against the disease.

Several studies indicated that farmers are more inclined to adopt biosecurity and disease control measures when they perceive them as important [44,45]. In another study, it was found that perceived strategy efficacy played a predominant role in the adoption of animal disease management strategies, especially in the context of biosecurity measures [46]. We assumed that if pigkeepers perceived certain measures as ineffective or unfavourable, their compliance with those measures would likely be lower; conversely, measures they favoured and found effective would likely have higher compliance rates. The participants in our study were mostly aware of the basic principles of disease control, such as disinfection measures (named in various formulations by all focus groups), and the limited access of people to the farm or changing clothes and shoes before entering the pigs’ premises. Regarding disinfection measures, however, the participants could not distinguish between preventive measures and measures used to eradicate infection or for internal biosecurity. Disinfection of their pigs’ premises was highly ranked as the most effective preventive measure, although it does not prevent the introduction of the virus to the herd. Several FGs named measures not related to ASF prevention, such as manure removal, bedding change, and washing the pigs with water, which indicates that possibly the participants might not fully understand the question and the concept of ‘introduction of the disease.’ Participants in one group mentioned the use of vaccines as a preventive measure against ASF, asserting that they possessed an official document regarding the vaccination of their pigs against both CSF and ASF (although this document was not presented to the facilitator). This finding is in line with a study by Muñoz-Gómez et al. [36], in which 21.6% of respondents in a questionnaire study among smallholder pigkeepers marked vaccination as an available preventive tool against ASF. To our knowledge, no ASF vaccine was officially available in the market or used in Ukraine before or during the study period. The participants’ mention of an ASF vaccination was most likely attributable to their confusion with the regular vaccination efforts against CSF conducted by Ukrainian veterinary authorities on smallholder farms. None of the FGs listed fencing of farm perimeters as a preventive measure against ASF. A possible explanation for this could be that, in Ukraine, smallholdings in rural areas are typically surrounded by fences, and pig houses are located within these fenced areas. This is why the participants might not have considered this an extra biosecurity measure.

The implementation of preventive measures is likely influenced by farmers’ attitudes, which, in turn, are shaped by their belief in the effectiveness of the measures, the effort required, and the discomfort caused by their implementation. In our study, we employed the straightforward question ‘How do you like the measure?’ to assess their attitudes towards preventive measures. Our results show that measures needing less extra effort and resources were most favoured, such as an access ban for people, the minimisation of contacts between pigkeepers, and the heat treatment of feed. In contrast, cleaning and disinfection were less favoured. This indicates that veterinary authorities should pay special attention to these measures when they are explained to smallholders, and, if possible, incentives should be provided to ensure compliance with the requirements.

Successfully controlling a disease within an animal population is a collaborative effort that requires the involvement of multiple stakeholders. It is crucial that all the parties involved have a clear understanding of their respective roles and responsibilities within this framework. Trust between counterparts is also important to ensure the swift exchange of unbiased information. This, in turn, forms the foundation for informed decisions and actions. The results of our study revealed a low awareness among smallholder pigkeepers regarding the stakeholders involved in ASF control in Ukraine. All focus groups mentioned veterinary professionals of various capacities, including those working for the government and private practices. Hunters were mentioned once, which could be explained by the insufficient interaction between hunters and pigkeepers regarding ASF control. Promoting a culture of cooperation by establishing clear communication channels and mechanisms for information exchange and mutual support among pigkeepers, including smallholders and other stakeholders, can improve disease surveillance and control in the country. These results highlight the need for extensive awareness-raising efforts among smallholder farmers regarding disease control systems in the country.

The evaluation of pigkeepers’ trust in various parties regarding their role in disease control revealed that smallholders generally have more trust in private entities (such as the pigkeepers themselves) and veterinary professionals (including local official veterinarians, local private veterinarians, and district veterinary hospitals) than in other governmental institutions (such as the central veterinary authority, village administration, and police). The concept of social identity, which encompasses a shared sense of group membership and values, has been found to be positively correlated with farmers’ trust in the government and their intention to report disease outbreaks [47]. The perception of not receiving compensation likely stems from the previous compensation system funded by local budget reserve funds, which was in place before the implementation of a resolution from the government [9] that stipulated the allocation of compensation funds from the state budget. Pigkeepers’ distrust towards the government’s ability to provide compensation, coupled with their propensity to neglect the registration of pigs—a prerequisite for securing compensation—may result in failed compliance with control measures. When an outbreak occurs, pigkeepers may be inclined to ignore control measures, recognising that they are unlikely to receive legal compensation. In the study by Cooper et al. [33], there were accounts of community members hiding pigs to prevent the culling of their herd due to the fear of insufficient compensation. Raising awareness among smallholder pigkeepers about the conditions for receiving compensation from the state could potentially improve their trust in the authorities. Enhanced communication and trust-building can lead to the increased effectiveness of control programs.

Acceptability of disease control measures by all parties involved is a crucial prerequisite for the successful implementation of measures and the effective control of diseases. Discussions centred around the feed destruction and culling of pigs on the farm and in the protection zone brought up emotional and strong opinions among pigkeepers. The emotional hardships faced by farmers and animal health workers resulting from control measures were discovered in the study by Cooper et al. [33], in which depopulation campaigns emerged as a dominant topic in discussions. Partial culling as a chosen ASF control measure in the country, with limited resources for compensation, demonstrated benefits to farmers and veterinary services in the study by Nga et al. [48]. As was suggested by Cooper et al. [33], the topic of human trauma arising from animal disease control measures is often overlooked, highlighting the need for greater global attention to the profound and far-reaching effects of stamping out strategies. Our findings emphasise the importance of continuous communication between stakeholders, providing detailed explanations concerning the reasons for existing disease control strategies and compensation rules.

The consequences that arise from pigkeepers being in ASF restricted zones were identified in this study as unfavourable, with the strongest adverse impact on the pigkeepers’ economy and psychological wellbeing. Psychosocial impacts of ASF on farmers, along with the corresponding effects of control measures, were also found in the study by Cooper et al. [33]. This study’s findings on the precautionary slaughter for their own consumption and illegal sales of pigs align with previous studies, which reported that smallholder farmers, especially those in economically challenged areas, are inclined toward the sale or slaughter of pigs to minimize their economic losses [11,40,49]. Our results highlight the significance of providing attractive reporting incentives and fostering trust between authorities and smallholder pigkeepers for the early detection of and response to disease outbreaks.

The listed sources of information about ASF by participants show that pigkeepers consider digital and traditional sources of information essential depending on the context of its usage. Either veterinary authorities or veterinarians were mentioned by groups, indicating their importance in the dissemination of reliable information. Furthermore, groups emphasized the necessity of increased sources that provide comprehensive information on different aspects related to ASF.

Our study describes the perceptions and opinions of pigkeepers who voluntarily participated, using a convenience sampling method, which may not fully represent those who declined to participate. The total number of 52 participants may raise a question as to the representativeness of our study. However, in participatory studies a statistically representative sample size has not been considered necessary. The sample size is determined by the heterogeneity of the answers given by the participants and justified upon reaching a saturation of answers, meaning that no new information can be obtained from the participants through the addition of new focus groups [50]. As we reached saturation in participants responses, we could assume that this study is representative in regards of the smallholders in the study area. To ensure balanced group interactions and encourage open discussions involving everyone, it is important for the facilitator to have good communication skills and an understanding of power dynamics. This helps create an environment in which diverse viewpoints are welcomed and valued [51]. Hence, the potential for biased results stemming from imbalanced group dynamics cannot be completely dismissed. To address this issue, the facilitator included all perspectives in the discussion, especially during activities designed to reach consensus. This helped prevent data gaps among participants who might have been less dominant. Furthermore, our findings represent the views of pigkeepers with no experience with ASF outbreaks. Not having participants with previous experience may be considered a weakness of the study. However, this can also be seen as a strength, as the focus groups were more homogeneous and the potential dominance of more experienced participants was avoided. Moreover, incorporating the perspectives of pigkeepers without direct experience with ASF increases the likelihood of identifying starting points for early interventions.

## 5. Conclusions

- Smallholder pigkeepers in Ukraine generally have high awareness about ASF’s clinical features.
- There is a lack of knowledge about ASF transmission routes and, therefore, a limited understanding of preventive measures among pigkeepers.
- Smallholder pigkeepers in Ukraine acknowledge their role in disease control. Nevertheless, their trust in the government was relatively lower when compared to their trust in veterinarians. This trust disparity could create a barrier to effective collaboration during disease control efforts.
- In general, smallholder pigkeepers in Ukraine tended to accept the disease eradication measures implemented at the farm level during ASF outbreaks.
- Despite the existing compensation scheme for smallholder pigkeepers in Ukraine, the consequences of being in a zone with applied ASF restriction measures are seen as having a detrimental impact on pigkeepers’ economic prospects and personal well-being.
- Some measures applied in the ASF restricted zones were not accepted by smallholders in Ukraine. Therefore, it is important to provide detailed explanations to improve their understanding and increase their acceptance of these measures.
- While the use of web sources is increasing, veterinarians, traditional media, and spoken communication continue to play important role in the dissemination of information regarding ASF to smallholder pigkeepers in Ukraine. Additionally, pigkeepers emphasized the need for more comprehensive information from different sources on various aspects related to ASF. We suggest implementing various activities, including educational campaigns for pigkeepers, to address the challenge of ASF control in Ukrainian smallholdings.

## Figures and Tables

**Table 1:** Merging of listed items related to the veterinarian into a common notation.

N of Groups Naming the Listed Item	Listed Item	Notation of the Merged Item
2	Veterinarian	Veterinarian
1	Vet workers	

**Table 2:** Locations of ten focus group meetings and the number of participating pigkeepers.

Administrative Region ^a^	No. of Participating Pigkeepers	Gender Composition within the Group	
		Male	Female
Kyiv	5	1	4
Odesa	3	2	1
Cherkasy	4	2	2
Cherkasy	3	1	2
Sumy	7	0	7
Kyiv	5	0	5
Chernihiv	7	0	7
Vinnytsa	7	1	6
Zhytomyr	5	4	1
Rivne	6	2	4
Total	52	13	39

^a^ Highest administrative division of the country.

**Table 3:** ASF signs listed by pigkeepers and ranked according to their level of indicativeness.

Rank	Sign	*n* Groups Listing	Perceived Indicativeness (Score ^a^)
1	Fever	10	82.2
2	Skin haemorrhage	8	79.3
3	Loss of appetite	8	38.8
4	Lethargy	6	35.9
5	Difficulty standing (weakness)	3	16.0
6	Increased mortality	2	10.6
7	Enlarged lymph nodes	1	8.3
8	Lethargy, loss of appetite	1	6.4
9	Foam from the mouth	1	4.8
10	Diarrhea	1	3.8
11	Constipation	1	3.8
12	Red ears	1	2.3
13	Rickets	1	1.8
14	Swollen joints	1	0.0
14	Dyspnea	1	0.0
14	Head swelling	1	0.0

^a^ Sum of the weighted proportional piling scores.

**Table 4:** Transmission routes, as listed by pigkeepers, ranked according to the perceived risk of ASFV introduction to the herd.

Rank	Transmission Route	*n* Groups Listing	Perceived Risk of ASFV Introduction (Score ^a^)
1	Rodents	5	52.7
2	Shoes	6	43.6
3	Transport vehicles	7	34.4
4	Human	6	30.9
5	Feed	6	27.5
6	Air	6	24.6
7	Insects	3	23.1
8	Wild animals incl. wild boar	5	17.6
9	Clothes	2	12.7
10	Other domestic animals	3	11.1
11	Dust	1	10.1
12	Bought new young animals	1	8.9
13	Shoes, clothes	2	8.1
14	All animals	1	6.6
15	Birds	1	5.3
16	Semen	2	4.4
17	Meat washing water	1	3.6
18	Through the meat of infected pigs	1	2.7

^a^ Sum of the weighted proportional piling scores.

**Table 5:** Preventive measures listed by pigkeepers, ranked based on their perceived effectiveness in preventing ASF introduction to the farm, and described in terms of pigkeepers’ affinity towards their implementation.

Pigkeepers’ Affinity (Score ^a^)	Preventive Measure	*n* Groups Listing	Perceived Effectiveness (Score ^b^)
0.26	Disinfection of pig premises	9	56.5
0.96	Access ban for people	6	44.7
0.46	Changing clothes and shoes	6	27.2
0.73	Control of rodents and insects	3	25.4
1.00	Vaccine	1	16.7
−0.95	Manure removal	3	12.3
1.00	Changing bedding	2	11.8
1.00	Heat treatment of feed	1	9.7
0.00	Disinfection mats	4	8.4
1.00	Minimization of contact between pigkeepers	1	6.9
−0.46	Cleaning and disinfecting of troughs	2	6.8
1.00	Keeping pigs in outdoors enclosures for fresh air and sunny weather	1	5.8
0.50	Washing of pigs with water	2	5.8
1.00	Hygienic procedures for humans (showering, sauna)	1	4.7
0.00	Disinfection of pig premises, disinfection mats	1	4.0
0.40	Treatment of pigs against mites (kerosene)	1	3.6
1.00	Keeping feed with limited access to rodents	1	2.9
0.67	All-in-all-out management system	1	2.2

^a^ Average emoji score; ^b^ sum of the weighted proportional piling scores.

**Table 6:** Stakeholders in ASF control, listed by pigkeepers and ranked by the perceived role of each stakeholder and trust regarding their capability to carry out their role in ASF control.

Trust (Score ^a^)	Stakeholder	*n* Groups Listing	Perceived Role (Score ^b^)
8.8	Pigkeepers	10 (6/4) ^c^	118.0
4.6	Central veterinary authority	5	54.3
7.7	District veterinary hospital (district veterinary office)	3	38.2
7.0	Local private veterinarian	3	32.5
4.8	Village administration	6	17.5
10	Local official veterinarian	2	16.8
2.0	Ministry of Emergency Situations	3	15.7
4.4	Police	4	9.5
5.0	Professional hunters	1	6.4
4.0	Sanitary station	2	6.4
1.7	District Administration	3	6.1
3.0	Veterinary laboratory	1	3.6
0.0	District waste management service	1	2.0
10	Military	1	0.0

^a^ 10-bead scoring tool; ^b^ sum of the weighted proportional piling scores; ^c^ independently/after the suggestion of the facilitator.

**Table 7:** The acceptance of farm-level ASF eradication measures by pigkeepers.

On-Farm Eradication Measure	Acceptance (Score ^a^)
Cleaning and disinfection of the farm	1
Farm quarantine	0.9
Culling of all pigs on the farm	0.4
Destroying the feed and bedding materials on the farm	0.3

^a^ Average emoji score.

**Table 8:** The acceptance of the ASF restriction measures applied in restricted zones among pigkeepers.

ASF Restriction Measure Applied in Zones	Acceptance (Score ^a^)
Restrictions on trading live pigs and pork products in the protection and surveillance zones	9.0
Restrictions on moving pigs in the protection zone	8.8
Culling of all pigs in the protection zone	4.9

^a^ 10-bead scoring tool.

**Table 9:** Consequences of being in a zone with ASF restriction measures applied, listed by pigkeepers and ranked by their perceived impact on pigkeepers.

Rank	Consequence	*n* Groups Listing	Perceived Impact (Score ^a^)
1	Economic loss	7	104.0
2	Psychological stress	6	68.1
3	Precautionary slaughter of pigs for consumption	7	52.3
4	Illegal sales of pigs	1	8.3
5	Discomfort of being in the quarantine zone	1	6.7
6	Prohibition on keeping pigs	1	5.0
7	Prohibition on trading pork products	1	4.0
8	Forced disinfection of pig premises	1	1.7
9	Alcoholism	1	0.0
9	More free time	1	0.0
9	Death of pigs	1	0.0
9	You can’t buy a pig right away	1	0.0

^a^ Sum of the weighted proportional piling scores.

**Table 10:** Sources of information about ASF, listed by pigkeepers and ranked by their importance to pigkeepers.

Rank	Source of Information	*n* Groups Listing	Perceived Importance (Score ^a^)
1	Web search (Google)	8	126.8
2	Veterinary authority	3	64.7
3	Traditional media (television, radio)	9	51.5
4	Spoken communication (word-of-mouth news)	8	46.5
5	Veterinarian	3	44.8
6	Digital social networks (Viber, Facebook)	2	19.4
7	Posters, leaflets, brochures	3	13.6
8	Internet videos (Youtube)	2	8.9
9	Local newspaper	3	6.5
10	Specialized literature	1	5.0
11	Governmental websites	1	3.3

^a^ Sum of the weighted proportional piling scores.

## Data Availability

The original data used for the analyses can be obtained from the corresponding author after approval by the responsible institutions in Ukraine and Estonia.
